# Understanding Your Baby: protocol for a controlled parallel group study of a universal home-based educational program for first time parents

**DOI:** 10.1186/s40359-022-00924-3

**Published:** 2022-09-22

**Authors:** Mette Skovgaard Væver, Marianne Thode Krogh, Anne Christine Stuart, Eva Back Madsen, Tina Wahl Haase, Ida Egmose

**Affiliations:** Department of Psychology, University of Copenhagen, Øster Farimagsgade 2A, Building 03-2-216, 1353 Copenhagen K, Denmark

**Keywords:** Parenting education, Primary intervention, Community health services, Early intervention, Postnatal care, Father-child relations, Home visiting, Early childhood mental health

## Abstract

**Background:**

Infant mental health represents a significant public health issue. The transition to parenthood provides optimal opportunities for supporting parenting competence. Especially parental mentalization, i.e. the caregiver’s ability to notice and interpret the child’s behavior in terms of mental states, is important in infancy where the caregiver-infant communication is based solely on the infant’s behavioral cues.

**Methods:**

This study evaluates the efficacy of the intervention Understanding Your Baby (UYB) compared to Care As Usual (CAU) in 10 Danish municipalities. UYB aims at promoting parental competence in new parents by supporting them in noticing their infants’ behavioral cues and interpreting them in terms of mental states. Participants will be approximately 1,130 singletons and their parents. Inclusion criteria are first-time parents, minimum 18 years old, living in one of the 10 municipalities, and registered in the Danish Civil Registration Register (CPR). Around 230 health visitors deliver the UYB as part of their routine observation of infant social withdrawal in the Danish home visiting program. During an interaction between the health visitor and the infant, the health visitor articulates specific infant behaviors and helps the caregivers interpret these behaviors to mental states. The study is a controlled parallel group study with data obtained at four time points in two phases: First in the control group receiving the publicly available postnatal care (CAU), secondly in the intervention group after UYB implementation into the existing postnatal services. The primary outcome is maternal competence. Secondary measures include paternal competence, parental stress, parental mentalizing, and infant socioemotional development. Analysis will employ survey data and data from the health visitors’ register.

**Discussion:**

Results will provide evidence regarding the efficacy of UYB in promoting parenting competences. If proved effective, the study will represent a notable advance to initiating the UYB intervention as part of a better infant mental health strategy in Denmark. Conversely, if UYB is inferior to CAU, this is also important knowledge in regard to promoting parenting competence and infant mental health in a general population.

*Trial registration* https://ClinicalTrials.gov with ID no. NCT03991416. Registered at 19 June 2019—Retrospectively registered, https://clinicaltrials.gov/ct2/show/NCT03991416

**Supplementary Information:**

The online version contains supplementary material available at 10.1186/s40359-022-00924-3.

## Background

Infant and early childhood mental health represents a significant public health issue. Extensive research has shown that early adversity and exposure to early childhood stress are significant risk factors that may have detrimental long-term developmental consequences for the affected children. Negative outcomes are seen on a range of areas such as physical and mental health, educational and labor market success, social network, and establishing of family [1, 2]. A recent review including studies from Europe and the US estimated that 17% of children under six years of age experience mental health problems, with more than half of these children experiencing severe problems [3]. In line with this, a recent Danish report on mental health in children aged 0–9 years finds that around 16% of children experience mental health problems and by the age of 10 years, 8% have minimum one psychiatric diagnosis [4]. During the first year of life, parents are the best providers of nurturing care, which is why mental health promotion and indicated interventions aimed at parents are important [5]. The transition to parenthood provides optimal opportunities for reaching families, and first times parents are often eager to develop their parenting skills.

It is well known that the transition to parenthood represents a challenging time for all mothers and fathers, who need to adjust to the new roles and develop new skills and competencies [6]. Parenting self-efficacy or parental competence can be defined as the degree to which parents feel competent and confident in handling child problems [7] and is seen as a key factor in promoting healthy functioning for parents and their children [8]. Previous studies have shown that the development of parental competence is important not only for parental mental health but also in terms of parental behavior and ultimately child development (for a review, see [8]). Engagement between parents and their infant, expressed before speech, develops through cuddling, eye contact, smiles, vocalizations, and gestures; it is the engine that propels brain development. Through these mutually enjoyable interactions, parent and infant create a communication channel through which the infant develops language, forms cognitions, and comes to know the world around them [5].

Recent studies have suggested that a central aspect of parental competence is parental mentalization, i.e. the caregiver’s ability to notice and interpret the child’s behavior in terms of mental states, such as emotions, intentions, and needs [9, 10]. Numerous studies have shown that parental mentalization promotes infant development such as socioemotional development [9, 11, 12] and child attachment [13, 14]. Parental mentalization is especially important in infancy where the caregiver-infant communication is based solely on the infant’s behavioral cues [14]. By observing their young child and discerning their child’s needs and intentions, parents help them learn about the world by describing and explaining their own and their child’s behavior.

As such, there is ample of evidence for supporting new parents abilities to interpret and understand their infant’s behavioral cues. The aim of the Understanding Your Baby (UYB) intervention is to support the development of parental competence in new parents by aiding them in the process of noticing their infants’ behavioral cues and interpreting them in terms of mental states. The UYB is a universal program delivered by municipality health visitors in the context of the existing Danish home-visiting program and the newly implemented systematic screening for infant social withdrawal with the Alarm Distress Baby Scale (ADBB; [15]).

### The Danish home-visiting program and the ADBB observation as the context for the UYB

The Danish national guidelines for infant and child healthcare were issued by the Danish Health Authority in 2011 and are currently under revision [16]. Infant and child healthcare is delivered by health visitors in primary care at the municipal level. The health visitors are nurses with 1.5 years of additional training who work with health promotion and prevention at the community level [16]. During the child’s first year, health visitors provide their services almost exclusively through home visits conducted by the same health visitor, who thereby develops a trustful relationship with the families. The Danish Health Authority recommends there should be five or six home visits during the first year of the child’s life, but each municipality is free to decide the number (anywhere from two to eight) [16]. The service in the home visiting program is voluntary for the families, but statistics show that it is widely used and highly accepted by parents; 98–99% of all Danish families receive regular home visits by a health visitor in the infant’s first year of life [16]. At each visit, the health visitor measures the growth of the baby and advises parents about their infant’s physical and nutritional needs, and in recent years there has also been an increasing focus on infant social and emotional development. As a result, health visitors in more than 80% of Danish municipalities (79 out of 98 municipalities) have by now been trained and certified in the use of the ADBB.

The ADBB is a tool for systematic observation and identification of persistent social withdrawal in infants aged 2–24 months as an indicator of emotional distress in infants and young children [15]. Infant social withdrawal is associated with longitudinal emotional and behavioral problems [17, 18] as well as impaired cognitive [19–21] and language development [20, 22]. The ADBB consists of eight items focusing on different aspects of infant social behavior, such as eye contact, facial expression, vocalizations, and activity level. Each item is rated on a scale from 0–4, with higher scores indicating less optimal social behavior. Studies have shown that a total score of five or above is considered the optimal cutoff for social withdrawal [15, 23–25]. ADBB is an observer-rated screening tool with ratings being based on a brief interaction (3–15 min) between the observer and the child [15].

A recent implementation study showed that health visitors in the capital municipality of Denmark generally held a positive attitude towards the ADBB: Many health visitors experienced that by using the ADBB, their own focus on child social and emotional development was sharpened, and they developed a more precise and nuanced professional language for talking about early risk with the parents [26]. However, during ADBB courses the health visitors have expressed a need for more extensive training and a systematic tool to support their ability to describe the infant’s socioemotional cues and behavior during the ADBB and thereby share knowledge with the parents about the early socioemotional needs of the infant. This has motivated the development of the UYB intervention, which was developed by the research team (the authors of this paper) in collaboration with a group of health visitors from the participating municipalities. When children score above cutoff on the ADBB and/or there are other concerns for the wellbeing of the child and/or the family, families should receive an indicated intervention targeting the family and child’s needs. In contrast, the purpose of the UYB program is to provide psychoeducation to all first time parents about their infants’ socioemotional development and behavioral cues at different ages during the first year of life as well as to offer educational support regarding three overall themes: infant crying, screen time in the family, and ‘good enough’ parenting. The UYB program is delivered as an integrated component of the home visiting program (see later detailed description of the content of UYB).

### The efficacy of universal home-based interventions for new parents

The Danish national guidelines for infant and child healthcare comprise a program aimed to monitor infant and child health and development as well as to advise parents. The program is not a specific parenting program, as supervising regarding parenting is just one element in the overall universal surveillance program. The effect of the Danish surveillance program is at this point not very well documented, which is also the case of general surveillance programs internationally [4]. An international review studied the effect of health visitors conducting home visits, however for the majority of the studies included, the home visits were related to premature birth, health or social issues [27]. Based on the review, it was concluded that no negative effects of home visiting were reported. Positive outcomes included improvement in children’s mental development, mental health and physical growth, reduction in severity of maternal depression, improvement in maternal employment, education, nutrition and other health habits, and government cost saving. Three American studies of the Nurse Family Partnership (NFP) interventions found positive long-term effects of health visitors home visits on children’s mental health at 4, 6, and 9 years [28–30].

Universal approaches to parenting support have as their general aim to enhance the quality of the early family environment in the population at large as opposed to targeting a specific problem in identified cases of families and children with special needs [31]. A number of studies have focused on different universal parenting interventions, but the majority of these interventions have been delivered as a group format. For instance, in a review of the effects of universal parenting interventions for parents of infants, children and adolescents, Salari & Enebrink included 34 studies [32]. Of these, 17 were exclusively group based, 10 were based on a combination of group sessions and individual telephone consultations, five consisted of both individual and group sessions, one consisted of online sessions, and only one study evaluated the effect of a home-based intervention. Similarly, in a systematic review of the effects of parenting interventions universally offered to parents with infants, Pontoppidan et al. included seven studies of which four were exclusively group based, two consisted of both home visits and group sessions, and only one consisted of home-based visits. Overall, the evidence for the effect of universal parenting group programs was mixed [33].

In the two above-mentioned reviews, the one intervention study consisting exclusively of home-based visits was the same, namely the study described by Aronen, Arajärvi & Linnansaari, Aronen, Aronen & Kurkela, Teerikangas et al., and Aronen & Arajärvi [34–38]. In this Finnish study, the sample was randomly selected out of all those Helsinki families in which a child was born between July 1, 1975, and June 30, 1976 (5,500 families). Initially, eight maternity clinic districts were chosen, representing different social classes and living conditions in Helsinki; of the 5,500 families of the total cohort, 1,600 families lived in these areas. In the next step, every eighth family was picked, 196 families were invited, and a final group of 160 families consented to participate. The intervention consisted of a psychiatric nurse who visited half of the 160 participating families (the intervention group) approximately 10 times per year during the first five years of the child’s life. During the visits, the nurse talked with the parents about the child’s development and needs with the aim of modifying the parents’ child-rearing attitudes and practice by increasing their understanding of the child [36]. The results of this study have shown both short- and long-term positive effects of the counselling in both low- and high-risk families. The authors have conducted a number of follow-up studies on the children and found that when the children were 5 years old there were fewer children with mental health issues in the intervention group [34]. Likewise, when the children were 14–15 years old, the adolescents in the intervention group scored significantly lower on measures of psychiatric symptoms [36].

While the intervention in the Finnish study was quite extensive with 10 home visits a year in 5 years, other less extensive interventions have also shown an effect. Dodge et al. evaluated a universal, home-based intervention in which nurses delivered a manualized 4–7 session program to improve infant mental health and well-being [39]. The results of this study showed that mothers in the intervention group reported more positive parenting behaviors and lower rates of anxiety, and the quality of the home environments was also rated higher for the intervention group compared to the control group. However, no significant differences in negative parenting behaviors, knowledge of infant development, sense of parenting competence, father-infant relationship quality, or blinded in-home observer ratings of parenting quality were found.

Shorey et al. evaluated the effectiveness of a postnatal psychoeducational program in enhancing maternal parental self-efficacy and social support and reducing postnatal depression among first-time mothers [40]. The postnatal psychoeducation program consisted of a 90-min face-to-face educational session during the home visit, an educational booklet and three follow-up phone calls (about 30 min, delivered weekly up to 6 weeks post-delivery). The topics covered in the session included physical and psychological challenges after birth, the importance of family dynamics, and means of enhancing self-efficacy and help-seeking behaviors. The results showed that the intervention group had significantly higher scores of maternal parental self-efficacy and social support and lower scores of postnatal depression at 6 and 12 weeks postpartum compared to the control group.

In the context of preventing obesity, Adams et al. evaluated a homebased intervention focusing on reducing screen time and television exposure and promoting parent-infant interactive play as part of parental responsiveness [41]. Trained nurses delivered the intervention to mothers during home visits when the infants were 3–4, 16, 28, and 40 weeks old, and at a research center visit when the infants were 1 and 2 years old. As part of the general guidance regarding prevention of overweight, the nurses informed the parents about the American Academy of Pediatrics (AAP) recommendations regarding screen time. The results of the study showed that the children in the intervention group had less daily screen time and television exposure than the children in the control group, meaning that more infants in the intervention group met the AAP guidelines for daily screen time at 44 weeks and 1 year of age. Furthermore, the television was turned on fewer hours per day for the intervention group, and fewer mothers in the intervention group reported that the television was ever on during infant meals at 44 weeks, 1.5 years, and 2.5 years. There were no group differences regarding the frequency of interactive play.

At this point, a limited number of studies have focused on developing and evaluating the efficacy of universal, home-based parenting interventions, however the results from the existing studies look promising. Taken together, the results suggest that it may be possible to enhance parental self-efficacy and parenting competences even by using interventions that are rather low in intensity. However, the small number of studies focusing specifically on this type of intervention also point towards that more and larger controlled studies are needed in this area.

### Purpose of the study

The purpose of the current study is to evaluate the efficacy of the newly developed UYB program in 10 Danish municipalities. More specifically, the aim of the study is to test whether Care As Usual (CAU) including the UYB program in comparison to CAU alone will lead to:

1. Improved maternal competence (primary outcome)
2. Improved paternal competence (secondary outcome)
3. Improved parental mentalizing, i.e. reflective functioning and mind-mindedness (secondary outcome)
4. Reduced parental stress (secondary outcome)
5. Improved infant socioemotional development (secondary outcome)
6. Reduced infant social withdrawal (secondary outcome)
7. Reduced parental media use and infant screen time (secondary outcome)
8. Increased information about infant socioemotional development (secondary outcome)
9. Heterogeneity of effects across family types with more disadvantaged families and families met with more intensive UYB implementation gaining more from participation in the intervention (tertiary outcome)

## Methods

### Study design

The study is designed as a controlled parallel group study with two arms, with the control group of parents receiving the existing publicly available postnatal care services in the 10 municipalities, i.e. Care As Usual (CAU), and the intervention group of parents receiving the Understanding Your Baby (UYB) integrated in to the existing publicly available postnatal care services (i.e. CAU). Both groups come from the same 10 municipalities. The CAU group is included in the project’s first phase and when data collection for this group is finished, all health visitors in the 10 municipalities are trained in delivering the UYB intervention. When the health visitors have finished their training, the UYB group is included in the project’s second phase.

### Study setting

The study is conducted in collaboration with the health visitors in 10 municipalities across Denmark. A letter containing detailed information about the study is send to all eligible parents’ personal electronic mail box, and all questionnaires completed by the parents are filled out electronically. All participants give informed written consent before inclusion.

The 10 municipalities are invited to participate based on logistical reasons: In the 10 participating municipalities, all health visitors are trained and certified in the ADBB and before entering this study, the ADBB has been implemented for a minimum of three months in the municipality. The 10 municipalities differ in size and population, as shown in Table 1. The number of health visitors in each municipality vary between 10 and 48. All in all, around 230 health visitors will be trained in and deliver UYB in the study.

**Table 1:** Background information for the 10 municipalities

	Area (km^2^)	Number of inhabitants	% of DK's population (%)	% of immigrants and descendants (%)	% of people aged 15–69 with a middle or higher formal educated (%)	% of people aged 15–69 with a long or higher formal education (%)	Average income before tax, etc. ($)	% of fulltime unemployed in the workforce (%)	Number of live-born infants (2020)	Average age of first-time mothers	Average age of first-time fathers
*Municipality*											
Aalborg	1,137	221,114	3.77	11.05	22.70	8.85	49,494	4.6	2,355	29.0	30.8
Frederiksberg	9	103,782	1.77	19.30	37.98	21.61	65,621	4.4	1,522	31.7	33.1
Holbæk	577	72,662	1.24	10.26	15.07	3.95	52,285	2.9	616	28.6	30.1
Hvidovre	23	53,277	0.91	21.18	18.36	7.19	52,239	4.4	599	29.8	32.1
Høje-Taastrup	78	52,702	0.90	31.96	15.48	5.79	50,243	5.7	602	29.3	31.4
Køge	258	61,706	1.05	12.92	15.05	4.84	54,791	3.6	603	29.5	31.4
Lolland	887	40,241	0.69	9.27	9.13	1.61	43,842	4.8	215	27.4	30.3
Middelfart	299	39,520	0.67	7.55	16.30	4.97	55,190	2.7	317	28.2	30.7
Nyborg	277	32,055	0.55	8.94	14.20	3.84	48,812	3.5	237	28.4	30.2
Næstved	677	83,650	1.43	9.51	14.30	3.15	50,414	3.5	715	28.2	30.5
Denmark	42,947	5,867,412	100	14.31	20.32	8.02	53,697	3.8	60,937	29.6	31.5

### Participants

#### Inclusion criteria

Parents living in one of the 10 municipalities participating in the project are invited to participate in the study. The parents have to be registered in the Danish Civil Registration Register (CPR) as parents to a newborn baby to be invited for the study. Mothers are invited if they are first-time mothers and if they are living on the same address as the child. Fathers/partners are invited if they have custody of the child or if they are living on the same address as the child, and they are invited regardless of whether they have children from previous relationships or not.

#### Exclusion criteria

Parents younger than 18 years, parents who are legally incapacitated, and parents with twins are not invited for the study. Fathers/partners who are not living in the same municipality as the child are also not invited for the study.

### The study interventions

During the project’s first phase, the participants will receive the existing publicly available postnatal care services (CAU) in the 10 municipalities and these may vary as described in Table 2. In the project’s second phase, the participants will receive the UYB intervention integrated into the existing publicly available postnatal care services in the 10 municipalities. The dose of UYB will therefore vary according to the variation in the existing postnatal care services (Table 2).

**Table 2:** Information on visits from the health visitor and ADBB observations in the 10 municipalities

	Number of visits from the health visitor 0–12 months postpartum	Infant's age (months) when ADBB is conducted	Number of ADBB observations in total	ADBB observation 2–3 months	ADBB observation 4–6 months	ADBB observation 8–10 months
*Municipality*						
Aalborg	5	2, 4–6, 8–10	3	X	X	X
Frederiksberg	5	4, 8	2		X	X
Holbæk	6	2, 4, 8	3	X	X	X
Hvidovre	7	2, 8–10	2	X		X
Høje-Taastrup	6	2–3, 4–6	3	X	X	
Køge	5	2–3, 4–6, 9–10	3	X	X	X
Lolland	7	2, 4, 6, 8	4	X	X	X
Middelfart	6	2, 4, 8	3	X	X	X
Nyborg	5	2, 8–10	2	X		X
Næstved	7	4	1		X	

ADBB = Alarm Distress Baby Scale

### Care As Usual

The Care As Usual (CAU) group of first time parents receives the existing publicly available postnatal care services in the 10 municipalities. The existing publicly available postnatal care services include home visits by health visitors throughout the infant’s first year. During the visits, health visitors measure and weigh the baby and guide and support the parents in a range of areas, such as breastfeeding, infant sleep, infant language, motor and physical development, and parental mental health. At some home visits, the health visitors also conduct an ADBB observation. The number of visits and the number of ADBB observations vary in the different municipalities but across municipalities, all first-time families are visited at least five times, and at least one ADBB observation is conducted during the infant’s first year. The five visits take place in the days following birth, when the infant is 2–4 weeks, 2–3 months, 4–6 months, and 8–10 months, while the ADBB observation is typically conducted at the 2–3 months and/or 4–6 months visit.

### The Understanding Your Baby intervention

In the study’s second phase, the experimental group of first time parents receives the UYB integrated into the existing publicly available postnatal care services, i.e. the amount of potential delivered UYB range from one to five in the 10 municipalities (see Table 2). The UYB is delivered by health visitors to all first-time parents during routine home visits.

The UYB is a universal educational parenting intervention aimed at supporting and promoting parents’ curiosity and knowledge about their infants’ socioemotional needs, ability to meet their infants’ needs, and thereby facilitating the development of a secure attachment and infant optimal socioemotional development. The UYB uses the routine ADBB observational situation as a standardized setting for a systematic sharing of knowledge and a dialogue with parents about their infants’ socioemotional cues, needs, and development in all families—i.e. this dialogue takes place in all cases—whether or not the ADBB observation gives rise to a concern regarding the infant’s socioemotional development. Although the primary purpose of the ADBB observation is early identification of persistent infant social withdrawal, the ADBB also provides an optimal context to guide caregivers about their child’s social and emotional development. During the interaction between the health visitor and the infant, the health visitor articulates specific infant behaviors and help the caregivers to interpret these behaviors in terms of mental states. For instance, when the infant looks away, the health visitor may explain to the parents that the infant needs a break to regulate themselves because interaction and eye contact is very stimulating for small children. In this way, the parents are shown their infant’s behavioral cues and how these behaviors are expressions of the inner world of the infant.

The UYB consists of four elements, each of which are described in the following: The UYB manual, the UYB dialogue cards, the UYB online video library, and UYB profiles on the social media platforms Facebook and Instagram.

The UYB manual entails an overview of research-based knowledge about infants’ socioemotional development and needs across four age groups (0–2, 2–4, 4–8, and 8–12 months) and five central behavioral areas inspired by the following items from the ADBB: Facial expressions (item 1), Eye contact (item 2), General level of activity (item 3), Vocalizations (item 5), Relationship and Attraction (items 7 and 8). In addition, the manual contains three chapters on significant themes for infant socioemotional development: infant crying, ‘good enough’ parenting, and screen time in the family. The manual provides the health visitors with the newest research-based knowledge on infant socioemotional development and enables them to provide research-based information to parents during the ADBB observations and when using the dialogue cards.

The UYB dialogue cards consist of four laminated cards, one that is mandatory and three that are optional for the health visitors to use. The mandatory UYB dialogue card is developed to support sharing of knowledge and facilitate reflection about the infant’s socioemotional cues and needs. The card displays the five areas from the ADBB also described in the manual. On the front of the card, short questions describe what the health visitor is observing when examining each of these areas, e.g. “What emotions does the baby express with his/her face?” (Facial Expressions). On the back of the card, short sentences describe how parents can support their infant’s development in relation to each of the areas, e.g. “Eye contact gives the baby a feeling of closeness and security. Breaks give the baby an opportunity to self-regulate. Notice when your baby seeks eye contact and when your baby needs a break.” This dialogue card should be used every time the health visitors conduct ADBB screenings in first-time families to inform parents about the specific areas related to infant social contact. Health visitors are also encouraged to use the card to provide feedback to parents about their infant’s behavior on each of these areas and guide parents in relation to how to support their child’s socioemotional development, also using age-specific and research-based knowledge from the manual.

The three optional UYB dialogue cards focus on infant crying, ‘good enough’ parenting, and screen time in the family. These cards may be used when health visitors believe that parents could benefit from talking about one of these topics. Each of these cards consists of a combination of research-based statements about the topic to increase parents’ knowledge, e.g. “Crying is the most important tool of the infant to express his/her needs and to achieve comfort and proximity form his/her parents”, and questions to facilitate reflection, e.g. “How is it for you when your infant is crying—what do you think and feel?”.

The UYB video library consists of videos categorized into the four age groups according to the manual, i.e. 0–2, 2–4, 4–8, and 8–12 months. The videos are designed to inform parents about their infant’s socioemotional needs and development, for example the development of the social smile (0–2 months), turn-taking (2–4 months), and social referencing (8–12 months). All videos contain clips of real parent-infant interactions exemplifying the socioemotional needs described by the speaker, e.g. an infant taking a break from the eye contact and the parent responding by reducing the amount of stimulation. Only examples of positive parenting behaviors are included in the videos. All videos are freely available online at the project homepage (www.forstaadinbaby.dk). It is a mandatory part of the UYB that health visitors inform first-time parents about the online video library (orally or via visiting cards). Health visitors are also encouraged to watch a video together with parents or groups of parents when possible.

Likewise, the health visitors may inform the parents about the UYB social media platforms. On the social media platforms, parents can find research-based knowledge about infant socioemotional development and parenting related to the first year of life. The same content is posted on Facebook and Instagram. Content are posted on the profiles regularly (typically twice a week), and both pictures and snippets of videos from the UYB video library are posted. The posted content is based on the research described in the UYB manual. In addition to posting research-based knowledge, the followers are engaged by Q&A’s (questions and answers) where they get an opportunity to ask questions about a specific topic, e.g. parent–child attachment, and then answers to selected questions are posted on the profiles the following weeks. The followers are also given a place to share their own experiences related to parenting by asking them questions and sharing their answers anonymously, e.g. “When are you most stressed as a parent?”. The aim is to communicate the research-based knowledge in a way that makes sense for all parents, and concrete examples of how the results can be translated into parenting behaviors are provided whenever possible. The concept of ‘good enough’ parenting is integrated into many of the posts to prevent or diminish some of the negative effects that new parenting information could create among the followers, e.g. that the parents feel that they should be doing all the things they read about on the profile all the time.

### The UYB training program

Health visitors are trained in the UYB on a two-day course, followed by two supervisions lasting three hours each, and two brush-ups lasting 1.5–3 h. The two-day course includes lectures on infant socioemotional development according to the topics described in the UYB manual as well as an introduction to the dialogue cards. Furthermore, the health visitors practice the sharing of age-specific and research-based knowledge to parents by watching video clips of ADBB screenings with infants scoring both below and above cutoff on the ADBB. More specifically, for each of the five areas from the ADBB, the health visitors practice describing the following three themes in regard to the video clips: 1. Infant behavior and underlying needs, 2. How parents can support infant socioemotional development (including what they are already doing, and what they should continue with), 3. Why this area is important for infant longitudinal development (e.g. how breaks from eye contact are needed for the child to self-regulate and engage in eye contact again). Finally, health visitors are introduced to the video library. After the two-day course, the health visitors should start using the UYB during their routine home visits.

Approximately two and four weeks after the course, the health visitors attend two three-hour group supervision sessions. During these supervisions, the implementation of the UYB in the health visitor’s daily practice is discussed and the health visitors watch more video clips of ADBB screenings. The purpose of the supervisions is for the health visitors to continue practicing their use of the mandatory dialogue card and articulating the infant’s behaviors as expressions of needs according to each of the five areas described on the card and in the manual.

At the two-day course and the two supervisions, the health visitors’ attendance is registered. Completion of the UYB training requires min. 80% attendance to the training and supervisions.

Around five months after the second group supervision, the health visitors attend the first brush-up session, which has the same format and content as the three-hour group supervisions. Finally, the health visitors attend a second (online) 1.5–2 h brush-up. This brush-up takes place approximately 5–9 months after the first brush-up and focuses on the health visitors discussing their daily practice and use of the UYB.

### Measures including outcomes

Different types of data are collected throughout the project: Data from questionnaires completed by parents and health visitors and data from the health visitors’ register.

The questionnaire data from parents and health visitors are collected and managed using REDCap electronic data capture tools hosted at University of Copenhagen [42, 43]. The parents are send questionnaire packages when their children are 2, 4, 7, and 11 months old, and it is possible to complete the questionnaires in both Danish and English. In a lottery, three gift cards are drawn each month, and the parents have the chance of winning a gift card worth approximately 76$ from a store selling baby items each time they answer one of the questionnaire packages. All participants are send a small present for the child, a children’s book worth approximately 6$, if they complete all four questionnaires. The health visitors are send questionnaires 1, 3, 6, and 12 months after they have finished the second UYB supervision. The health visitors are not offered any rewards for answering the questionnaires.

The data from the health visitors’ register is obtained after the children have turned 12 months. Data from this register is only obtained if at least one of the parents has given consent.

### Background information and control variables

*Background information* about the parents is obtained as part of the 2- and 11-months questionnaire. At 2 months, this information includes questions about parental age, gender, ethnicity, educational background, employment status, income, relationship status, smoking, persons living in the household, the birth weight of the child, birth complications, and siblings to the child. At 11 months, parents are asked about breastfeeding, parental leave, and parental involvement with the child.

*Parental depressive symptoms* will be measured using the Edinburgh Postnatal Depression Scale (EPDS; [44]) or the Gotland Male Depression Scale (GMDS; [45]). The EPDS is a screening instrument for postnatal depression, which has primarily been validated in female samples. The EPDS contains 10 items assessing the presence of depressive symptoms in the past seven days (e.g. “I have felt sad and miserable”). Each item is rated on a four-point scale from “No, not at all” (= 0) to “Yes, quite a lot” (= 3). GMDS is a screening tool for male depression and consists of 13 items assessing whether the respondent’s behavior has changed during the last month (e.g. “More aggressive, outward-reacting, difficulties keeping self-control”). Each item is rated on a 4-point Likert type scale from “Not at all” (= 0) to “Extremely so” (= 4) [46]. For both questionnaires, items are summed to a total score with higher scores reflecting higher levels of depressive symptoms. These questionnaires are routinely given to parents typically around 8 weeks postpartum as part of the health visitors’ mental health screening program. The parents fill in the questionnaires during the health visitor’s visit in either a web-based or paper-and-pencil manner. The EPDS and GMDS data will be obtained from the health visitors’ register.

*Intensity of UYB implementation* will be measured as a combination of data obtained from the health visitors’ register and questions asked to the parents about the different UYB elements. The data obtained from the register will be the number of ADBB-screenings during the first year of life, and whether the health visitor was certified in UYB at the time of the screening. The questions asked to the parents include number of home visits during the first year of life, and whether they have seen the dialogue card(s), followed UYB on social media, or used the video library. The questions regarding the number of home visits, the dialogue card(s), and the social media will be asked at 11 months while the questions regarding the video library will be asked at 2, 4, 7, and 11 months.

### Primary study outcome

*Parenting competence* will be measured using the Parenting Sense of Competence Scale (PSOC; [47, 48]). In the current study, the Danish version of the PSOC translated by Lange et al. [49] is used. The most commonly used tool for measuring parental competence is the PSOC [50]. The PSOC was originally developed by Gibaud-Wallston in 1977, and the scale has subsequently been revised and used in a number of studies (for instance, [51–53]). The PSOC consists of 17 items rated on a 6-point Likert type scale from “Strongly agree” to “Strongly disagree”. The scale has two subscales, with eight of the items being summed together to calculate the Skill/Knowledge subscale and the remaining nine items being summed together to create the Valuing/Comfort subscale. A total score is calculated by summing all 17 items (of which nine are reverse-scored), with higher scores indicating a higher level of parenting competence [54]. Parenting competence is measured at 2, 4, 7, and 11 months.

### Secondary study outcomes

*Parental reflective functioning* will be measured using the Parental reflective functioning questionnaire (PRFQ; [14]), a self-report measure of the parental ability to reflect upon the mental states of oneself and the child. In this study, we use the 15-item version of the PRFQ, which has been validated in a sample of Danish mothers of infants [55]. The items are rated on a 7-point Likert type scale going from “Completely disagree” (= 1) to “Completely agree” (= 7). Items are summed together to form three subscales measuring different aspects of parental mentalizing: (a) Interest and curiosity in the child’s mental states (e.g. “I like to think about the reasons behind the way my child behaves and feel”), (b) Certainty about the child’s mental states (e.g. “I can completely read my child’s mind”), and (c) Pre-mentalizing modes, i.e. difficulties recognizing the child’s mental states (e.g. “My child cries around strangers to embarrass me”). Parental reflective functioning is measured at 4, 7, and 11 months.

*Parental representational mind-mindedness* will be assessed using the written response to the following open-ended question: “We would like you to describe your child using your own words. You can write exactly what comes to mind, and you can write as little or as much as you like.” The response is used to code parental mind-mindedness, i.e. the ability to generate mental descriptions to the child in writing. Parental mind-mindedness is coded based on the guidelines described in [56]. To control for opacity, mind-mindedness is calculated as the number of mental descriptors divided by the total number of attributes used. Parental mind-mindedness is measured at 2, 4, 7, and 11 months.

*Parenting stress* will be measured using the Parenting Stress Index, Fourth Edition – short form (PSI-4-SF; [57]), a 36-item questionnaire measuring stress in relation to being a parent. Each item is rated on a 5-point Likert type scale from “Strongly disagree” (= 1) to “Strongly agree” (= 5). The PSI-SF provides a total score and three subscales: (a) Parental distress, measuring stress related to the parent’s experience of parenting and/or his or her parenting abilities (e.g. “I feel trapped by my responsibilities as a parent”), (b) Parent–child dysfunctional interaction, measuring stress related to the parent’s experience of interactions with his or her child (e.g. “I expected to have closer and warmer feelings for my child than I do, and this bothers me”), and (c) Difficult child, measuring stress related to the parent’s experience of his or her child’s characteristic (e.g. “My child makes more demands on me than most children”). Parenting stress is measured at 4 and 11 months.

*Infant socioemotional development* will be measured using the Ages & Stages Questionnaires®: Social-Emotional, Second Edition (ASQ:SE-2; [58]). The ASQ:SE-2 questionnaire for children aged 9 to 14 months contains 27 items assessing the presence of different socioemotional behaviors (e.g. “Does your baby smile at you and other family members?”). There are three choices for each item: “Most of the time” (= 0), “Sometimes” (= 5), and “Rarely or never” (= 10). In addition, the parent can check a “concern” option for each item, which adds 5 points to the score for that item. Items are summed to a total score with higher values reflecting less optimal socioemotional development. Empirically derived cutoffs are available to determine whether the child’s socioemotional development appears to be on schedule, in the monitor zone, or in need for further evaluation [58]. Infant socioemotional development is measured at 11 months.

*Infant social withdrawal* will be measured using the Alarm Distress Baby Scale (ADBB; [15]). The ADBB is an observer-rated screening tool consisting of eight items: Facial expressions, Eye contact, General level of activity, Self-stimulating gestures, Vocalizations, Briskness of response to stimulation, Relationship, and Attraction. Each item is rated on a 5-point scale from “Absolutely normal” (= 0) to “Very obvious abnormal behavior” (= 4). Items are summed to a total score with higher scores indicating more social withdrawal. A cutoff of ≥ 5 may be used to identify infants showing clinical levels of social withdrawal [15, 23–25]. The ADBB data will be obtained from the health visitors’ register and will be based on the ADBB observations performed by the health visitors at the home visits (see Table 2).

*Parental media use and infant screen time* will be measured using a short questionnaire developed by the authors focusing on the use of different types of screens (television, tablets, smartphones, etc.) in the families (Additional file 1). The questions address the parent’s screen time during the infant’s awake time (total minutes per day), the infant’s screen time (total minutes per day), and the parent’s perception of technoference in the parent–child relationship (number of disturbances per day). Media use is measured at 2, 4, 7, and 11 months.

*Information about infant development* is assessed at 11 months using a short questionnaire developed by the authors (Additional file 2). Parents are asked to what degree they have been informed about each of the following topics by their health visitor during their infant’s first year: (a) My child’s physical development, (b) My child’s nutrition and sleep, (c) My child’s emotional development, (d) How I manage my child’s emotions, (e) My child’s social competences and development, and (f) How I support my child’s social development. In this study, we are interested in whether parents have received more information about the last four topics.﻿ 

Table 3 shows an overview of all the questionnaires that are completed by the parents at the different measurement points.

**Table 3:** Points of measurements of control variables, primary and secondary outcomes

Measures	T1 (2 months)	T2 (4 months)	T3 (7 months)	T4 (11 months)	Obtained from the health visitors’ register
*CAU and UYB*					
Background information, incl. Socioeconomic status (SES)	X			X	
Depressive symptoms (EPDS and GMDS)					X
Parenting Sense of Competence Scale (PSOC)	X	X	X	X	
Parental Reflective Functioning Questionnaire (PRFQ)		X	X	X	
Parental Mind Mindedness (MM)—written description	X	X	X	X	
Parenting Stress Index, Fourth Edition—short form (PSI-4-SF)		X		X	
Ages and Stages Questionnaire®: Socio-Emotion, Second Edition (ASQ:SE-2)				X	
Alarm Distress Baby scale (ADBB)					X
Parental media use and infant screen time	X	X	X	X	
Information about infant development				X	
*Only UYB*					
Intensity of UYB implementation				X	

*CAU* Care As Usual. *UYB* Understanding Your Baby

### Sample size

We used the R package simr [59] to conduct a power analysis. We wanted to be able to detect a small effect size (Cohen’s *d* = 0.2; [60]) using a linear mixed model with one predictor (i.e. group status) and three covariates (all continuous). Using a power of 0.80 and a significance level of 0.05, 690 participants are needed in order to detect a small effect. Assuming that 25% of the participants drop out after intake and before the endpoint, a total of 920 participants will have to be recruited. A participant is considered to have dropped out when they actively withdraws from the study or does not answer any of the questionnaires.

### Statistical methods

The analyses of both primary and secondary outcomes will be conducted under the intention to treat principle where the participants are analysed according to their control CAU/intervention UYB status. However, as a secondary analysis, we will also conduct analyses where the actual intervention participation is taken into account (i.e. whether the families in the UYB group have been given the intervention by the health visitors). Thus, we will perform a contamination adjusted intention to treat analysis [61] which compliments the intention-to-treat approach by producing a better estimate of the effect of the UYB intervention.

The primary endpoint is PSOC at 11 months postpartum. The level of significance will be 5%, two-tailed [62]. The primary comparison of UYB and CAU interventions is analysed using linear mixed models with the health visitor as the second order variable. This is done to account for the correlation induced by the same health visitor conducting the CAU and UYB intervention for multiple families. We will control for maternal educational level in years, maternal level of depressive symptoms, and maternal age in years.

Secondary outcomes will be analysed in the same way as the primary outcome. Binary and categorical outcomes will be analysed with mixed logistic and multinomial logistic regressions, respectively, and continuous outcomes with mixed linear regression analyses. For outcomes where we are interested in the individual variance across time points, generalized estimating equations will be employed. All the analyses will be adjusted for the abovementioned covariates. As multiple tests will be conducted, we will both assess significance using the 5% significance level and adjust for multiple testing using the Bonferroni-Holm method [63].

For the tertiary outcome, we investigate whether some subpopulations benefit more from the UYB intervention. These subpopulations will be defined as disadvantaged families (e.g. based on background information such as employment status, income, etc.) versus advantageous families as well as families with a more intensive UYB implementation. In practice, we will test for significant subpopulations-group status interaction effects using linear moderation analyses (as otherwise described above).

Regarding missing data, the first step will be to delete the group status variable to be blinded. If more than 95% of the observations are complete, a complete case analysis will be conducted. Otherwise, data-driven, pragmatic choices will be made in selecting a few background variables (e.g. educational level, parental age at birth, level of depressive symptoms) to see which ones are strong predictors of missing status and then use multiple imputation to handle missing data [64]. Imputations will be conducted using the software package ‘mice’ [65].

### Dates defining periods of recruitment and follow-up

The project’s first phase, i.e. recruitment of the CAU group, began May 15th, 2019 and ended February 29th, 2020. This group includes parents and their infants born from April 1st until September 30th, 2019 in the 10 municipalities. In August 2020, the health visitors began training in the UYB-intervention. However, due to COVID-19, the training of the health visitors in three of the 10 municipalities had to be postponed and was finished by March 2021. In seven municipalities, the project’s second phase, i.e. the recruitment of the UYB group, will take place in the period from February 15th until November 30th, 2021. This group includes parents and infants born from January 1st until June 30th, 2021. In the three municipalities where the UYB training was delayed, the participant recruitment will take place in the period from May 15th, 2021 until February 28th, 2022. This group includes parents and infants born from April 1st, 2021 and until September 30th, 2021. Data collection will go on until December 2022.

### Harms

The UYB is a universal educational parenting intervention aimed at supporting first time parents and delivered by municipality health visitors within the context of the ADBB observation and the already existing postnatal services. Participation in the study is voluntary and declining to participate does not in any way affect access to family services provided by the municipality. For these reasons, we expect the intervention to be associated with very low risk for the participants.

### Registration numbers and name of trial registry and ethical approval

The project is registered at ClinicalTrials.gov with ID no. NCT03991416. The project has gotten an ethical approval by the Institutional Review Board at the Department of Psychology, University of Copenhagen, with approval number IP-IRB/23112018 (23/11–2018).

## Discussion

The aim of the Understanding Your Baby (UYB) intervention is to support the development of parental competence in new parents by helping them in the process of noticing their infants’ behavioral cues and interpreting them in terms of mental states. The UYB is a universal program delivered by municipality health visitors in the context of the existing Danish home-visiting program and the newly implemented systematic screening for infant social withdrawal with the Alarm Distress Baby Scale. The protocol describes a controlled parallel group study designed to evaluate the efficacy of UYB in comparison to existing municipality postnatal services. At this point, a limited number of studies have focused on developing and evaluating the efficacy of universal, home-based parenting interventions, however the results from the existing studies look promising. Universal approaches to parenting support have as their general aim to enhance the quality of the early family environment in the population at large, and findings from previous studies suggest that it may be possible to enhance parental self-efficacy and parenting competences even by using interventions that are rather low in intensity.

Previous studies on the effect of universal parenting interventions have shown small to medium effects [32, 33], therefore the present study is designed to be able to capture a small effect of the UYB. According to the “prevention paradox” coined by epidemiologist Geoffrey Rose [66], although a preventive universal intervention may bring little benefit to each person, the cumulative consequence can still be a large benefit to the community, whereas an indicated intervention bringing large benefits to each person may have a small impact on the community. Following this, it becomes important to minimize the effort and potential harms arising from universal interventions [66, 67]. Since the UYB is integrated into the already existing Danish home-visiting program, attending the intervention does not require any additional effort from the parents, and apart from the health visitors attendance at the training, it does not require any additional resources from the municipalities. Furthermore, examining whether UYB is inferior to CAU on any of the outcomes provide important insight into any potential harms of the intervention. Based on this, we argue that even a small positive effect of UYB represent a notable advance to initiating the intervention as part of a better infant mental health strategy in Denmark.

As described previously, the data collection for the study started in May 2019, with the data for the CAU group being collected between May 2019 and September 2020 and the data for the UYB group being collected between February 2021 and December 2022. With the first Danish patient testing positive for COVID-19 in February 2020 and the first national lockdown beginning in March the same year, the data collection for the CAU and UYB groups has taken place during a period where COVID-19 lockdowns and restrictions have affected the everyday lives of most adults and children in Denmark. However, since the first lockdown came while the data collection for the CAU group was well under way, it was not possible to implement any new measures in the study that could have been used to measure the influence of COVID-19 on the sample or to measure COVID-19 related differences between the CAU and the UYB group. Hence, an unforeseen but important limitation of the study will be that the COVID-19 lockdowns and constantly changing number of restrictions may have affected the study in a number of ways. Since the restrictions have varied over time, participants in the two groups and participants in each group may have been affected differently by COVID-19, and it cannot be ruled out that the pandemic have affected the primary or secondary outcomes of the study. Furthermore, the heath visitors’ delivery of both CAU and the UYB intervention may also have been negatively affected by the pandemic.

Nonetheless, results from this study will provide new evidence regarding the efficacy of UYB in the 10 municipalities. To the extent that the UYB shows to be a promising approach, health visitors in other municipalities may be trained in UYB in a future up-scaling.

## Supplementary Information

**Additional file 1** Parental media use and infant screen time. This file contains the questionnaire about parental media use and infant screen time

**Additional file 2** Information from the health visitor about infant development. This file contains the questionnaire about the information that the parents have gotten from the health visitor about infant development

## Data Availability

Not applicable.

## Abbreviations

AAP: American Academy of Pediatrics
ADBB: Alarm Distress Baby Scale
ASQSE-2: Ages and Stages Questionnaire®: Socio-Emotional, Second Edition
CAU: Care As Usual
CPR: Danish Civil Registration Register
DK: Denmark
EPDS: Edinburgh Postnatal Depression Scale
FAD: McMaster family assessment device
GF6 +: General functioning scale
GMDS: Gotland Male Depression Scale
MM: Mind Mindedness
NFP: Nurse Family Partnership
PRFQ: Parental Reflective Functioning Questionnaire
PSI-4-SF: Parenting Stress Index, Fourth Edition—short form
PSOC: Parenting Sense of Competence Scale
SES: Socioeconomic status
UYB: Understanding Your Baby
