# Supplementary material for: Understanding Your Baby: protocol for a controlled parallel group study of a universal home-based educational program for first time parents
Source: BMC Psychol. 2022 Sep 22;10:223. doi: 10.1186/s40359-022-00924-3 (PMC9502638; doi:10.1186/s40359-022-00924-3)
Supplement: Supplementary file 1 — Additional file 1 Parental media use and infant screen time. This file contains the questionnaire about parental media use and infant screen time [file 40359_2022_924_MOESM1_ESM.docx]

Additional file 1

**Parental media use and infant screen time questionnaire**

1. On a typical day, when your child is awake, how often is the television, a tablet, a smartphone, or another type of screen also turned on?

1. Never or almost never

2. A smaller part of the time

3. Half the time

4. A large part of the time

5. All the time or almost all the time

1. On a typical day, how many times does your time together with your child get interrupted because you look at your telephone, the television, a tablet, or another type of screen?

1. 0-1 times a day

2. 2-3 times a day

3. 4-6 times a day

4. 7-10 times a day

5. 11-15 times a day

6. 16-20 times a day

7. More than 20 times a day

1. On a typical day, how many times does your partner’s time together with your child get interrupted because he/she looks at the telephone, the television, a tablet, or another type of screen? (If you do not have a partner, please answer "Not relevant")

1. 0-1 times a day

2. 2-3 times a day

3. 4-6 times a day

4. 7-10 times a day

5. 11-15 times a day

6. 16-20 times a day

7. More than 20 times a day

8. Not relevant

1. On a typical day, how many minutes or hours does your child look at the television, a smartphone, a tablet, or other types of screens?

1. Less than 5 minutes a day

2. 5-15 minutes a day

3. 15-30 minutes a day

4. 30-60 minutes a day

5. 1-1½ hours a day

6. 1½-2 hours a day

7. 2-3 hours a day

8. 3-4 hours a day

9. More than 4 hours a day
