# Supplementary material for: Understanding Your Baby: protocol for a controlled parallel group study of a universal home-based educational program for first time parents
Source: BMC Psychol. 2022 Sep 22;10:223. doi: 10.1186/s40359-022-00924-3 (PMC9502638; doi:10.1186/s40359-022-00924-3)
Supplement: Supplementary file 2 — Additional file 2 Information from the health visitor about infant development. This file contains the questionnaire about the information that the parents have gotten from the health visitor about infant development [file 40359_2022_924_MOESM2_ESM.docx]

Additional file 2

**Information from the health visitor about infant development questionnaire**

1. Please rate the extent to which you feel that you have been informed about the following subjects by your health visitor during your child's first year:

1. My child's physical and motor development

2. My child's nutrition

3. My child's sleep

4. My child's social and emotional development

5. How I support my child's social and emotional development

Each subject is rated using the following scale:

1. Not at all

2. To a smaller degree

3. To some degree

4. To a large degree

5. Don't know

1. Please rate the extent to which you feel that you have been missing information from your health visitor about the following subjects during your child's first year:

1. My child's physical and motor development

2. My child's nutrition

3. My child's sleep

4. My child's social and emotional development

5. How I support my child's social and emotional development

Each subject is rated using the following scale:

1. Not at all

2. To a smaller degree

3. To some degree

4. To a large degree

5. Don't know
